# Proprioceptive Sensors’ Fault Tolerant Control Strategy for an Autonomous Vehicle

**DOI:** 10.3390/s18061893

**Published:** 2018-06-09

**Authors:** Mohamed Riad Boukhari, Ahmed Chaibet, Moussa Boukhnifer, Sébastien Glaser

**Affiliations:** 1 Institut VEDECOM, 77 Rue des Chantiers, Versailles 78000, France; 2 ESTACA, 12 Rue Paul Delouvrier, Montigny-le-Bretonneux 78180, France; ahmed.chaibet@estaca.fr (A.C.); moussa.boukhnifer@estaca.fr (M.B.); 3 Centre for Accident Research and Road Safety (CARRS-Q), Queensland University of Technology (QUT), Brisbane City, QLD 4000, Australia; sebastien.glaser@qut.edu.au

**Keywords:** sensor fault, fault tolerant control, autonomous vehicle, separation principle, Lipschitz model, LMI approach

## Abstract

In this contribution, a fault-tolerant control strategy for the longitudinal dynamics of an autonomous vehicle is presented. The aim is to be able to detect potential failures of the vehicle’s speed sensor and then to keep the vehicle in a safe state. For this purpose, the separation principle, composed of a static output feedback controller and fault estimation observers, is designed. Indeed, two observer techniques were proposed: the proportional and integral observer and the descriptor observer. The effectiveness of the proposed scheme is validated by means of the experimental demonstrator of the VEDECOM (Véhicle Décarboné et Communinicant) Institut.

## 1. Introduction

The development of autonomous vehicles is enjoying a huge infatuation among the scientific community around the world. Hence, the research projects are globally intended to reduce traffic accidents, cut down fuel consumption and increase the efficiency of transportation systems, as well as passengers’ safety. In fact, vehicles’ automated systems have seen a significant advancement in a few decades, and several commercial cars are equipped with smart ADAS systems. Additionally, some leading companies around the world have shown promising results of fully-autonomous driving vehicles, such as the Google Car, Cruise of GM and the Tesla Autopilot. From this perspective, the SAE On-Road Automated Vehicle Standards Committee assessedr the classification of autonomous vehicles [1]. The Information Report provides a taxonomy for motor vehicle automation ranging in level from no automation to full automation. Thus, high levels of automation (namely 4 and 5) exclude the driver as a fall-back solution and must operate under specific environments (Level 4) or in all possible situations (Level 5), by ensuring autonomous monitoring of the surrounding area, the performance of the sensors and the algorithms and by deciding on the actions to maintain the vehicle in a safe state. Consequently, the monitoring of the performance of vehicle sensors is critical, as long as the entire operation of the car depends deeply on the sensors’ information. Indeed, the sensors’ reliability is a serious concern, especially since the architecture of an autonomous vehicle includes the tasks of: perception, localization, planning, control and system management, which share information with each other [2]; that is why a single faulty task can result in the dangerous behaviour of the vehicle.

From this perspective, it is necessary to design a fault tolerant control mechanism that can diagnose faults and preserve a safe vehicle behaviour even under faulty or degraded sensing. Furthermore, the designed fault tolerant mechanism must meet the requirements of the complexity of the high vehicle dynamics.

The fault tolerant control field has been an active research area. It is based on the inter-connection between the domains of system modelling, control theory and fault diagnosis. Several techniques and methodologies have been inventoried in review papers [3,4,5,6,7,8,9,10,11,12,13,14,15,16,17,18,19,20,21]. Mainly, there are two major branches of fault tolerant control, techniques that need a system analytical model and techniques that do not. Hence, methods without models are simple to synthesize and implement. On the other have, they have some issues in the event of transient faults, where the controlled closed loop system tends to dampen the effects of faults, and so, simply checking the size of the output signals does not give reliable insight into the overall system health [22]. Advances in the control theory field have boosted the development of mathematical modelling, made possible with recent computer technology. Hence, the model-based techniques provide a significant robustness against exogenous disturbances and structural model uncertainties, thanks to the $H_{\infty }$ and Lyapunov techniques. Further, exploiting analytical dynamic relations leads to estimated variables that cannot (or their dedicated sensors are expansive) be measured [23].

Moreover, looking at road death casualties, the human driver is mostly responsible, and the automated system may, hypothetically, decrease the number of deaths on the road by 90% [24]. Further, the report of the French Inter-ministerial Office of Road Safety pointed out that speeding was one of the main causes of accidents in French roads for the year 2015 with about 32% of the cases. Indeed, speed control and regulation are critical in such automated systems [25]. Hence, the automated vehicle must have the ability to adjust its speed keeping, and, subsequently, a safe distance behind the front vehicle. This safe distance is a sufficient condition to immediately stop the vehicle if the front target stops rapidly, avoiding the collision. Thus, it is clear that inaccuracies or faults in the vehicle speed sensor can corrupt the inter-distance, leading to injurious control actions [26]. The problem of the vehicle speed sensor reliability belongs to the general family of proprioceptive fault diagnosis for which several techniques have been presented in the literature. The main methodologies aim to take advantage of the model-based techniques, mainly because of their robustness. Main methodologies aim to take advantage of the Model-Based techniques, mainly because of their robustness. In fact, the fault diagnosis task can be performed by using a large panel of methods, such as the parameter estimation (mainly for process faults), that exploit the the input/output pair to estimate the process model, and judge later whether the estimated parameters are more or less close to the parameters of the nominal case [27]. An other manner to proceed, is the design of a residual generator matrix using parity equations. Thus, the generated residuals give indications of the health of the diagnosed system, in such a way that if they are null it implies that there is no fault, contrariwise, if they are non null, one can conclude that some faults occurred [28]. Both aforementioned techniques can diagnose a fault, but they lack in robustness by leading to false alarms, due to the fact that they have a threshold, and the robust design of one threshold ensuring a global operating range is a harsh task. Thus, fault estimation observers appear to be an advantageous tool. Additionally, the reduced order observers can estimate states which dedicated sensors are very expensive, and can thereby, replace this expensive sensors. Several techniques of observers were presented in the literature recently, thus, it can be cited non exhaustively: the Unknown Input Observer (UIO) [29,30,31], Disturbance Observer [32,33], Descriptor Observer [34,35,36], Kalman Filtering variants [37,38,39]. As aforementioned, the main purpose of this work is to counteract the sensor faults of an autonomous vehicle. The proposed scheme in this paper aims to overcome the proprioceptive additive faults, with its three different forms (abrupt fault, intermittent fault and incipient fault) [40], as they occur with a far higher probability than multiplicative faults [41]. Furthermore, it is assumed in the designed scheme that there are no occurrence of parametric (tire deflation) and actuator (motor abnormalities) faults when the vehicle is operating.

The remainder of this paper is divided into the following sections: Section 2 describes the problem that we will be undertaking and the theoretical methods, while the experiments are described in Section 3. Section 4 shows the experimental results and discussions. Concluding remarks of this contribution are given in Section 5.

## 2. Materials and Methods

**Figure 1:**
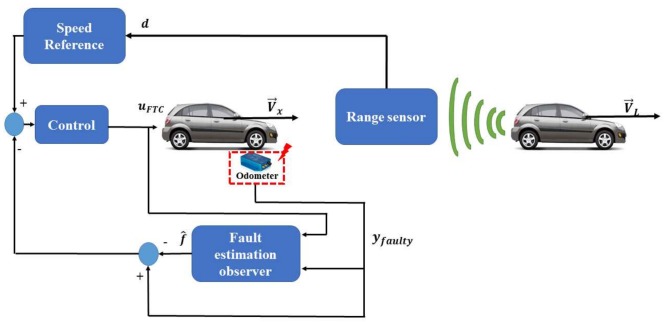
Adaptive Cruise Control (ACC) fault tolerant paradigm.

To tackle the probable proprioceptive sensor faults, an active fault tolerant strategy based on the separation principle is designed. The FTC scheme consists of: a Static Output Feedback controller (SOF) and a fault estimation observer. Hence, the SOF ensures the tracking of the reference speed, and it is designed in the nominal case (faultless scenarios). The aim of the separation principle is to keep the controller running always in the nominal case, even if faults are occurring. This is made possible by subtracting the estimated fault from the measurement signal, in such a way that the controller uses a healthy speed signal (see Figure 1). The proper functioning of the proposed scheme is ensured by the robustness of the controller and the perfect fault estimation provided by the observer [23].

In this study, we are interested in designing a controller and two observers for the vehicle longitudinal dynamics in the Adaptive Cruise Control (ACC) driving scenario. Furthermore, the single track representation is advantageous in ensuring simplicity. The vehicle dynamics relies on the following assumptions [42,43,44]:

- The road is assumed to be a plane (no slope, no inclination);
- The lateral dynamics is not considered;
- Yaw, pitch and roll dynamics are neglected.

**Figure 2:**
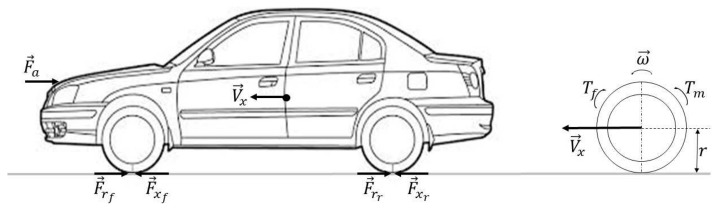
Vehicle longitudinal dynamics.

Considering the above assumptions, the longitudinal vehicle dynamics can be expressed by the following equations (see Figure 2):

(1) $$\left\{\begin{array}{cc} m{\dot{V}}_{x}\left(t\right) & =\sum_{i=1}^{4}F_{x_{i}}\left(t\right)-F_{a}\left(t\right)\\ {\bar{J}}_{r_{i}}{\dot{\omega }}_{r_{i}}\left(t\right) & =T_{m}\left(t\right)-rF_{x_{i}}\left(t\right)-rF_{r_{i}}\left(t\right)-T_{b_{i}}\left(t\right),i=1,2\\ J_{r_{i}}{\dot{\omega }}_{r_{i}}\left(t\right) & =-rF_{x_{i}}\left(t\right)-rF_{r_{i}}\left(t\right)-T_{b_{i}}\left(t\right),i=3,4 \end{array}\right.$$

where the vehicle parameters are expressed in Table 1. Adopting a single track modelling by defining: $T_{b_{f}}=T_{b_{1}}+T_{b_{2}},T_{b_{r}}=T_{b_{3}}+T_{b_{4}},T_{r_{f}}=r\left(F_{r_{1}}+F_{r_{2}}\right),T_{r_{r}}=r\left(F_{r_{3}}+F_{r_{4}}\right),F_{x_{f}}=F_{x_{1}}+F_{x_{2}},F_{x_{r}}=F_{x_{3}}+F_{x_{4}},{\dot{\omega }}_{r_{f}}={\dot{\omega }}_{r_{1}}={\dot{\omega }}_{r_{2}},{\dot{\omega }}_{r_{r}}={\dot{\omega }}_{r_{3}}={\dot{\omega }}_{r_{4}},{\bar{J}}_{r}={\bar{J}}_{r_{1}}+{\bar{J}}_{r_{2}},J_{r}=J_{r_{3}}+J_{r_{4}}$

We obtain the following equation:

(2) $$\left\{\begin{array}{c} m{\dot{V}}_{x}\left(t\right)=F_{x_{f}}\left(t\right)+F_{x_{r}}\left(t\right)-F_{a}\left(t\right)\\ {\bar{J}}_{r}{\dot{\omega }}_{r_{f}}\left(t\right)=T_{m}\left(t\right)-rF_{x_{f}}\left(t\right)-T_{r_{f}}\left(t\right)-T_{b_{f}}\left(t\right)\\ J_{r}{\dot{\omega }}_{r_{r}}\left(t\right)=-rF_{x_{r}}\left(t\right)-T_{r_{r}}\left(t\right)-T_{b_{r}}\left(t\right) \end{array}\right.$$

Substituting $F_{x_{f}}$ and $F_{x_{r}}$ in Equation (1) leads to:

(3) $$\begin{array}{c} m{\dot{V}}_{x}\left(t\right)=\frac{1}{r}\left[T_{m}\left(t\right)-T_{b_{f}}\left(t\right)-T_{b_{r}}\left(t\right)-T_{r_{f}}\left(t\right)-T_{r_{r}}\left(t\right)-{\bar{J}}_{r}{\dot{\omega }}_{r_{f}}\left(t\right)-J_{r}{\dot{\omega }}_{r_{r}}\left(t\right)\right]-F_{a}\left(t\right) \end{array}$$

The longitudinal slip ratio hypothesis can be written as:

$$\lambda =\frac{r\omega_{r}-V_{x}}{max(r\omega_{r},V_{x})}=0$$

leading to $r\omega_{r}=V_{x}$, then $r{\dot{\omega }}_{r}={\dot{V}}_{x}$. Substituting ${\dot{\omega }}_{r}$ in (3), we obtain:

(4) $$\begin{array}{c} \left(m+\frac{{\bar{J}}_{r}+J_{r}}{r^{2}}\right){\dot{V}}_{x}\left(t\right)=\frac{1}{r}\left[T_{m}\left(t\right)-T_{b_{f}}\left(t\right)-T_{b_{r}}\left(t\right)-T_{r_{f}}\left(t\right)-T_{r_{r}}\left(t\right)\right]-F_{a}\left(t\right) \end{array}$$

Denoting $T_{b}=T_{b_{f}}+T_{b_{r}}$, $T_{r}=T_{r_{f}}+T_{r_{r}}$, we get:

(5) $$J_{eq}{\dot{V}}_{x}\left(t\right)=T_{eq}\left(t\right)-aV_{x}\left(t\right)-bV_{x}^{2}\left(t\right)$$

where $J_{eq}=\left(rm+\frac{{\bar{J}}_{r}+J_{r}}{r}\right)$, $T_{eq}=T_{m}-T_{f}-T_{r}$, $F_{a}=aV_{x}+bV_{x}^{2}$ and *a* and *b* are aerodynamic coefficients. $T_{eq}$ is the torque given by the engine and the brake system; its dynamics is subject to loss and to a decay rate. To overcome this problem, we assume a first order dynamics, with a time constant τ [26]:

(6) $${\dot{T}}_{eq}\left(t\right)=\frac{1}{\tau }\left(-T_{eq}\left(t\right)+u\left(t\right)\right)$$

Finally, the vehicle longitudinal dynamics is given in the quadratic form by combining (5) and (6) as follows:

(7) $$\begin{array}{c} \left[\begin{array}{c} {\dot{V}}_{x}\left(t\right)\\ {\dot{T}}_{eq}\left(t\right) \end{array}\right]=\left[\begin{array}{cc} \frac{-a}{J_{eq}} & \frac{1}{J_{eq}}\\ 0 & \frac{-1}{\tau } \end{array}\right]\left[\begin{array}{c} V_{x}\left(t\right)\\ T_{eq}\left(t\right) \end{array}\right]+\left[\begin{array}{c} 0\\ \frac{1}{\tau } \end{array}\right]u\left(t\right)+\left[\begin{array}{c} \frac{-b}{J_{eq}}\\ 0 \end{array}\right]V_{x}^{2} \end{array}$$

The model (7) can be written in the following Lipschitz nonlinear form:

(8) $$\left\{\begin{array}{cc} \dot{x}\left(t\right) & =Ax(t)+Bu(t)+Gg(x(t))\\ y(t) & =Cx(t) \end{array}\right.$$

with: $x=\left[\begin{array}{c} V_{x}\\ T_{eq} \end{array}\right]$, $A=\left[\begin{array}{cc} \frac{-a}{J_{eq}} & \frac{1}{J_{eq}}\\ 0 & \frac{-1}{\tau } \end{array}\right]$, $B=\left[\begin{array}{c} 0\\ \frac{1}{\tau } \end{array}\right]$, $G=\left[\begin{array}{c} \frac{-b}{J_{eq}}\\ 0 \end{array}\right]$, $g\left(x\right)=V_{x}^{2}$, $C=\left[\begin{array}{cc} 1 & 0 \end{array}\right]$.

Taking into account the exogenous disturbances:

(9) $$\left\{\begin{array}{cc} \dot{x}\left(t\right) & =Ax(t)+Bu(t)+Gg(x(t))+Wd(t)\\ y(t) & =Cx(t) \end{array}\right.$$

where d(t) is the disturbance signal and *W* the disturbance distribution matrix of the appropriate dimension.

In order to design the controller and the observers, we adopt the following hypothesis and algebraic lemmas:

**Hypothesis** **1.** *The nonlinear terms are considered to be a smooth Lipschitz function satisfying the following relation:*

(10) $$||g\left(x_{1}\right)-g\left(x_{2}\right)\left|\right|\leq l||x_{1}-x_{2}\left|\right|$$

*where $x_{1},x_{2}\in R^{n}$ and l is a positive Lipschitz constant.*

**Hypothesis** **2.** *The sensor faults $f_{s}\left(t\right)$ and the exogenous disturbances w(t) are assumed to be bounded:*

(11) $$\left\{\begin{array}{cc} \left|\right|f_{s}\left(t\right)\left|\right| & \leq f_{max}\\ \left||w(t)|\right| & \leq w_{max} \end{array}\right.$$

*where $f_{max}$ and $w_{max}$ are constant scalars.*

**Lemma 1** (Schur complement). *Given the matrices $S\in R^{n\times n}$, $M\in R^{n\times m}$ and $\Gamma \in R^{m\times m}$, the following implication holds [45]:*

(12) $$S+M\Gamma^{-1}M^{T}<0,\Gamma <0\Leftrightarrow \left[\begin{array}{cc} S & M\\ M^{T} & \Gamma \end{array}\right]<0$$

**Lemma** **2.** *Consider matrices A, B and a scalar δ; the following inequality holds [32]:*

(13) $$A^{T}B+B^{T}A\leq \delta A^{T}A+\delta^{-1}B^{T}B$$

**Lemma 3** (Elimination lemma). *Assume matrices $Q\in S^{n}$, $B\in R^{m\times n}$ and $C\in R^{p\times n}$. Thus, the following statements are equivalent [46]:*

- ($B^{{⊥}^{T}}QB^{⊥}<0$ or $B^{T}B>0$) and ($C^{{⊥}^{T}}QC^{⊥}<0$ or $C^{T}C>0$);
- $\exists \;K\in R^{p\times m}:Q+C^{T}KB+B^{T}K^{T}C<0$.

### 2.1. Static Output Feedback Controller Design

The controller must ensure a robust tracking of the reference signal against mismodelling dynamics and model noises. Indeed, for the sake of convenience with the separation principle, the SOF controller is designed in this subsection. In fact, this control technique is more appropriate with the longitudinal vehicle dynamics model. The output feedback theory is an active research area, and several works are presented in the literature [47]. These works aim to overcome the multiple challenges of the output feedback methodology.

In order to design the SOF controller, we rewrite the system (3) in the following form:

(14) $$\left\{\begin{array}{cc} \dot{x} & =Ax+Gg(x)+Bu+Ww\\ z & =C_{z}x+B_{z}u+W_{z}w\\ y & =Cx+W_{y}w \end{array}\right.$$

where *z* is the performance output vector, with $C_{z},\;B_{z}$ and $W_{z}$ being matrices of appropriate dimensions.

The stabilizable static output feedback controller is given by:

(15) $$u=-K_{SOF}y$$

The real problem of the static output feedback control design lies in the difficulty of designing the gain $K_{SOF}$ when the matrix *C* is singular (as in our case). To deal with this issue, the system parametrization of Lemma 4 is adopted [48].

**Lemma** **4.** *The parametrization of System (14) minimizing an optimal $H_{\infty }$ criterion $\gamma_{00}^{2}$ for a SOF controller is given by:*

(16) $$\begin{array}{cc} ℵ(P_{\infty }) & =\left[\begin{array}{cccc} \delta_{00}l^{2}{\bar{I}}_{00} & 0 & 0 & 0\\ 0 & 0 & 0 & 0\\ 0 & 0 & 0 & 0\\ 0 & 0 & 0 & -\delta_{00}^{-1}I \end{array}\right]+{\left[\begin{array}{cccc} A & W & B & G\\ I & 0 & 0 & 0 \end{array}\right]}^{T}\left[\begin{array}{cc} P_{\infty } & 0\\ 0 & P_{\infty } \end{array}\right]\left[\begin{array}{cccc} A & W & B & G\\ I & 0 & 0 & 0 \end{array}\right]\\ & +{\left[\begin{array}{cccc} C_{z} & B_{z} & W_{z} & 0\\ 0 & 0 & I & 0 \end{array}\right]}^{T}\left[\begin{array}{cc} I & 0\\ 0 & -\gamma_{00}^{2}I \end{array}\right]\left[\begin{array}{cccc} C_{z} & B_{z} & W_{z} & 0\\ 0 & 0 & I & 0 \end{array}\right] \end{array}$$

**Proof.**  Let us consider the following relation:

(17) $$\begin{array}{cc} J=: & V\left(P_{\infty }\right)+J\left(\delta_{00}^{-1}\right)\\ =: & x^{T}P_{\infty }x+\int_{0}^{\infty }\left(z^{T}z-\gamma_{00}^{2}w^{T}w\right)dt \end{array}$$

Deriving the relation (17) yields:

(18) $$\dot{J}=:{\dot{x}}^{T}P_{\infty }x+x^{T}P_{\infty }\dot{x}+z^{T}z-\gamma_{00}^{2}w^{T}w$$

Developing (18), by using the model (14), gives:

(19) $$\begin{array}{cc} \dot{J}=: & x^{T}A^{T}P_{\infty }x+u^{T}B^{T}P_{\infty }x+g^{T}\left(x\right)G^{T}P_{\infty }x+w^{T}W^{T}P_{\infty }x\\ & +x^{T}P_{\infty }Ax+x^{T}P_{\infty }Bx+x^{T}P_{\infty }Gg\left(x\right)+x^{T}P_{\infty }Ww+x^{T}C_{z}^{T}C_{z}x+x^{T}C_{z}^{T}B_{z}u\\ & +x^{T}C_{z}^{T}W_{z}w+u^{T}B_{z}^{T}C_{z}x+u^{T}B_{z}^{T}B_{z}u+u^{T}B_{z}^{T}W_{z}w+w^{T}W_{z}^{T}C_{z}x\\ & +w^{T}W_{z}^{T}B_{z}u+w^{T}W_{z}^{T}W_{z}w-\gamma_{00}^{2}w^{T}w \end{array}$$

Based on Hypothesis 1 and Lemma 2, the relation (19) can be written in the quadratic form of (20).

(20) $$\begin{array}{cc} \dot{J}=: & {\left[\begin{array}{c} x\\ u\\ w \end{array}\right]}^{T}\left[\begin{array}{ccc} A^{T}P_{\infty }+P_{\infty }A+\delta_{00}l^{2}{\bar{I}}_{00}+\delta_{00}^{-1}P_{\infty }GG^{T}P_{\infty } & P_{\infty }B & P_{\infty }W\\ B^{T}P_{\infty } & 0 & 0\\ W^{T}P_{\infty } & 0 & 0 \end{array}\right]\\ + & \left[\begin{array}{ccc} C_{z}^{T}C_{z} & C_{z}^{T}B_{z} & C_{z}^{T}W_{z}\\ B_{z}^{T}C_{z} & B_{z}^{T}B_{z} & B_{z}^{T}W_{z}\\ W_{z}^{T}C_{z} & W_{z}^{T}B_{z} & W_{z}^{T}W_{z}-\gamma_{00}^{2}I \end{array}\right]\left[\begin{array}{c} x\\ u\\ w \end{array}\right] \end{array}$$

Using the Schur complement, we obtain:

(21) $$\begin{array}{cc} \dot{J}=: & \left[\begin{array}{cccc} A^{T}P_{\infty }+P_{\infty }A+\delta_{00}l^{2}{\bar{I}}_{00} & P_{\infty }B & P_{\infty }W & P_{\infty }G\\ B^{T}P_{\infty } & 0 & 0 & 0\\ W^{T}P_{\infty } & 0 & 0 & 0\\ G^{T}P_{\infty } & 0 & 0 & -\delta_{00}^{-1}I \end{array}\right]\\ + & \left[\begin{array}{cccc} C_{z}^{T}C_{z} & C_{z}^{T}B_{z} & C_{z}^{T}W_{z} & 0\\ B_{z}^{T}C_{z} & B_{z}^{T}B_{z} & B_{z}^{T}W_{z} & 0\\ W_{z}^{T}C_{z} & W_{z}^{T}B_{z} & W_{z}^{T}W_{z}-\gamma_{00}^{2}I & 0\\ 0 & 0 & 0 & 0 \end{array}\right] \end{array}$$

The expression (16) can be obtained easily after that concluded from (21) by factorization, and that ends the proof. □

Using the compact form of parametrization lemma, the optimal control gain is then obtained by the following theorem:

**Theorem** **1.** *The system (14) is stabilizable by the static output feedback controller $K_{SOF}$, and minimizing a $H_{\infty }$ criterion $\gamma_{00}^{2}$, if there exist, a positive semidefinite matrix $P_{\infty }\in R^{2\times 2}$, matrices $Q_{00}\in R^{1\times 1}$, $Q_{01}\in R^{1\times 1}$, $K_{SF}\in R^{1\times 2}$, $K_{w}\in R^{1\times 2}$ and scalars $\delta_{00}$ and $\gamma_{00}$, such that the following constraints are satisfied [48]:*

(22) $$\begin{array}{c} \min_{\begin{array}{c} P_{\infty },Q_{00},Q_{01},K_{w},K_{SF}\\ subject\;to \end{array}}\gamma_{00}^{2}\\ P_{\infty }\geq 0,\\ ℵ\left(P_{\infty }\right)+H\left(\begin{array}{cc} \left[\begin{array}{c} K_{SF}^{T}\\ K_{w}^{T}\\ -I\\ 0 \end{array}\right] & \left[\begin{array}{cccc} Q_{00}C & Q_{00}W_{y} & Q_{01} & 0 \end{array}\right] \end{array}\right)\leq 0 \end{array}$$

The static output feedback controller can finally be deduced by: $K_{SOF}=-Q_{01}^{-1}Q_{00}$.

**Proof.**  Taking advantage of the parametrization lemma (Lemma 4), the closed loop formulation of the static output feedback $H_{\infty }$ control problem can be written as follows:

(23) $$\begin{array}{c} \min_{\begin{array}{c} P_{\infty },K_{SOF}\\ subject\;to \end{array}}\gamma_{00}^{2}\\ P_{\infty }\geq 0,\\ {\left[\begin{array}{cc} I & 0\\ 0 & I\\ -K_{SOF}C & -K_{SOF}W_{y}\\ I & 0 \end{array}\right]}^{T}ℵ\left(P_{\infty }\right)\left[\begin{array}{cc} I & 0\\ 0 & I\\ -K_{SOF}C & -K_{SOF}W_{y}\\ I & 0 \end{array}\right]\leq 0 \end{array}$$

By application of Lemma 3, we obtain:

(24) $$\begin{array}{c} \min_{\begin{array}{c} P_{\infty },K_{SOF},F_{\infty 1},F_{\infty 2},Q_{01}\\ subject\;to \end{array}}\gamma_{00}^{2}\\ P_{\infty }\geq 0,\\ ℵ\left(P_{\infty }\right)+H\left(\begin{array}{cc} \left[\begin{array}{c} F_{\infty 1}^{T}\\ F_{\infty 2}^{T}\\ -Q_{01}\\ 0 \end{array}\right] & \left[\begin{array}{cccc} K_{SOF}C & K_{SOF}W_{y} & I & 0 \end{array}\right] \end{array}\right)\leq 0 \end{array}$$

Factorizing the matrix $Q_{01}$ in (24) (Note that for this purpose, the matrix $Q_{01}$ must be invertible. This exigence is verified, since the block (3,3) of (24) reads $Q_{01}+Q_{01}^{T}\;<\;0$.), we get:

(25) $$\begin{array}{c} \min_{\begin{array}{c} P_{\infty },K_{SOF},F_{\infty 1},F_{\infty 2},Q_{01}\\ subject\;to \end{array}}\gamma_{00}^{2}\\ P_{\infty }\geq 0,\\ ℵ\left(P_{\infty }\right)+H\left(\begin{array}{cc} \left[\begin{array}{c} Q_{01}^{T}F_{\infty 1}^{T}\\ Q_{01}^{T}F_{\infty 2}^{T}\\ -I\\ 0 \end{array}\right] & \left[\begin{array}{cccc} Q_{01}K_{SOF}C & Q_{01}K_{SOF}W_{y} & Q_{01} & 0 \end{array}\right] \end{array}\right)\leq 0 \end{array}$$

LMI summarised in Relation (22) can be easily deduced from Relation (25), by taking the following notations: $K_{SF}^{T}=Q_{01}^{T}F_{\infty 1}^{T}$, $K_{w}^{T}=Q_{01}^{T}F_{\infty 2}^{T}$, $Q_{00}=Q_{01}K_{SOF}$. □

**Remark** **1.** *It is clear that Theorem 1 is a non-convex optimization problem, and its solution seems to be non trivial. To overcome this issue, an initialization of the variables $K_{w}$ and $K_{SF}$ can be obtained reasonably by solving the following $H_{\infty }$ optimal problem [48]:*

(26) $$\begin{array}{c} \min_{\begin{array}{c} X_{\infty },K_{\infty 1},K_{\infty 2},Y,\delta_{01}\\ subject\;to \end{array}}\gamma_{01}^{2}\\ X_{\infty }\geq 0,\\ \left[\begin{array}{cccc} H\left(AX_{\infty }-BY\right)+\delta_{01}l^{2}{\bar{I}}_{01} & W+BK_{\infty 1} & {\left(C_{z}+B_{z}K_{\infty 2}\right)}^{T} & G\\ {}* & -\gamma_{01}^{2}I & {\left(W_{z}+B_{z}K_{\infty 1}\right)}^{T} & 0\\ {}* & * & -I & 0\\ {}* & * & * & -\delta_{01}^{-1}I \end{array}\right]\leq 0 \end{array}$$

Finally, we obtain: $K_{SF}=YX_{\infty }^{-1}$ and $K_{w}=K_{\infty 1}$.

**Proof.**  For brevity, the proof is omitted here, but the reader can refer to [48] and the references therein. □

The cross-decomposition Algorithm 1 to design the optimal static output feedback controller is then given as follows:
**Algorithm 1** The cross-decomposition algorithm.
Initialization step (k=1): solve LMI (26) and choose $K_{SF}$ and $K_{w}$;Iterative step (*k*):
(a)first part: solve LMI of Theorem 1, and fix $Q_{01}$ and $Q_{00}$;(b)second part: solve LMI of Theorem 1, and fix $K_{SF}$;
Final step: if $\gamma_{00}^{part\;1}-\gamma_{00}^{part\;2}<\epsilon$ then choose $K_{SOF}=-Q_{01}^{-1}Q_{00}$, else k=k+1 and return to Step 2.

where ϵ is a desired performance determined by the designer.

### 2.2. Proportional and Integral Observer Design

The proportional and integral observer has been broadly developed and applied in recent years for the topic of fault diagnosis and fault tolerant control [49]. It has a strong ability in obtaining fault information, such as the size and the shape. Indeed, the additive sensor fault can be estimated for the faulty system of the following form:

(27) $$\left\{\begin{array}{cc} \dot{x} & =Ax+Gg(x)+Bu+Ww\\ y & =Cx+Ff_{s} \end{array}\right.$$

where $f_{s}$ is the additive sensor fault and *F* is the fault matrix distribution.

The proportional and integral fault estimation observer of the system (27) is described by [50]:

(28) $$\left\{\begin{array}{cc} \dot{\hat{x}} & =A\hat{x}+Gg\left(\hat{x}\right)+Bu+L_{p}\left(y-C\hat{x}-F\hat{f}\right)\\ {\dot{\hat{f}}}_{s} & =L_{I}\left(y-C\hat{x}-F\hat{f}\right) \end{array}\right.$$

To calculate the observer gains $L_{p}$ and $L_{I}$, we adopt the following considerations:

- The estimated state error *e* is defined as $e=x-\hat{x}$;
- The estimated fault error $e_{f}$ is defined as $e_{f}=f_{s}-{\hat{f}}_{s}$;
- The free fault case ($f_{s}=0$), residual signal *r*, is defined as $r=N_{1}\left(y-\hat{y}\right)=\left[\begin{array}{c} N_{1}\\ 0 \end{array}\right]\left[\begin{array}{cc} C & 0 \end{array}\right]\left[\begin{array}{c} e\\ e_{f} \end{array}\right]=N_{1}C_{0}\left[\begin{array}{c} e\\ e_{f} \end{array}\right]$ (where $N_{1}\in R^{2\times 1}$ is a weighting matrix to be designed).

By taking into account the latter considerations, the dynamics of estimated state error and estimated fault error are written:

(29) $$\left\{\begin{array}{cc} \dot{e} & =\left(A-L_{p}C\right)e+G\tilde{g}+Ww-L_{p}Fe_{f}\\ {\dot{e}}_{f} & ={\dot{f}}_{s}-L_{I}Ce-L_{I}Fe_{f} \end{array}\right.$$

where $\tilde{g}=g\left(x\left(t\right)\right)-g\left(\hat{x}\left(t\right)\right)$. Based on (28) and (29), an augmented system of the following form can be written:

(30) $$\left\{\begin{array}{cc} {\dot{Z}}_{1} & =\left(A-LC\right)Z_{1}+G\tilde{g}+W\tilde{w}\\ {\dot{Z}}_{2} & =AZ_{2}+LCZ_{1}+Gg\left(\hat{x}\right)+Bu \end{array}\right.$$

where:

(31) $$\begin{array}{ccc} Z_{1}\left(t\right)=\left[\begin{array}{c} e\\ e_{f} \end{array}\right] & Z_{2}\left(t\right)=\left[\begin{array}{c} \hat{x}\\ {\hat{f}}_{s} \end{array}\right] & A=\left[\begin{array}{cc} A & 0_{2\times 2}\\ 0_{2\times 2} & 0_{2\times 2} \end{array}\right] \end{array}$$

(32) $$\begin{array}{ccc} L=\left[\begin{array}{c} L_{p}\\ L_{I} \end{array}\right] & C=\left[\begin{array}{cc} C & F \end{array}\right] & B=\left[\begin{array}{c} B\\ 0_{2\times 1} \end{array}\right] \end{array}$$

(33) $$\begin{array}{ccc} G=\left[\begin{array}{c} G\\ 0_{2\times 1} \end{array}\right] & W=\left[\begin{array}{cc} W & 0_{2\times 2}\\ 0_{2\times 3} & \left[\begin{array}{c} 0\\ 1 \end{array}\right] \end{array}\right] & \tilde{w}=\left[\begin{array}{c} w\\ {\dot{f}}_{s} \end{array}\right] \end{array}$$

In order to make the residual signal *r* insensitive to external disturbances *w*, we propose the following property:

**Property** **1.** *The $H_{\infty }$ criterion ensuring the disturbances rejection is written as follows [51]:*

(34) $$\int_{0}^{\infty }\left(r^{T}r-\gamma^{2}w^{T}w\right)dt\leq 0$$

**Theorem** **2.** *The nonlinear Lipschitz Proportional and Integral Observer (28) is asymptotically stable, if there exist positive definite matrices $P_{1}$ and $P_{2}$, matrices $U_{1}$, $U_{2}$ and $N_{1}$ and positive scalars $\delta_{i},\left(i=1,2\right)$, λ and γ, such that the following LMI is verified:*

(35) $$\begin{array}{c} \left[\begin{array}{cc} \Sigma_{11} & \Upsilon_{11}\\ {}* & \Xi_{11} \end{array}\right]\leq 0 \end{array}$$

(36) $$\begin{array}{c} P_{1}^{-1}U_{1}=P_{2}^{-1}U_{2} \end{array}$$

*and:*

(37) $$\begin{array}{ccc} \Sigma_{11} & = & \left[\begin{array}{cc} A^{T}P_{1}+P_{1}A-C^{T}U_{1}^{T}+\delta_{1}l^{2}\bar{I} & C^{T}U_{2}^{T}\\ U_{2}C & A^{T}P_{2}+P_{2}A+I+\delta_{2}l^{2}\bar{I} \end{array}\right] \end{array}$$

(38) $$\begin{array}{ccc} \Upsilon_{11} & = & \left[\begin{array}{ccccc} 0 & P_{1}W & P_{1}G & C_{0}^{T}N_{1}^{T} & 0\\ P_{2}B & 0 & 0 & 0 & P_{2}G \end{array}\right] \end{array}$$

(39) $$\begin{array}{ccc} \Xi_{11} & = & \left[\begin{array}{ccccc} -\lambda^{2}I & 0 & 0 & 0 & 0\\ 0 & -\gamma^{2}I_{2} & 0 & 0 & 0\\ 0 & 0 & -\delta_{1}^{-1}I & 0 & 0\\ 0 & 0 & 0 & -I & 0\\ 0 & 0 & 0 & 0 & -\delta_{2}^{-1}I \end{array}\right] \end{array}$$

*Finally, the observer gains are calculated:*

(40) $$\begin{array}{c} L=P_{1}^{-1}U_{1} \end{array}$$

**Proof.**  Consider the following multiple Lyapunov function, where matrices $P_{1}=P_{1}^{T}$ and $P_{2}=P_{2}^{T}$ are symmetric definite positive matrices:

(41) $$\begin{array}{c} V=Z_{1}^{T}P_{1}Z_{1}+Z_{2}^{T}P_{2}Z_{2}>0 \end{array}$$

Deriving (41) and using Property 1 and $L_{2}$, based on Lyapunov theory, we obtain:

(42) $$\begin{array}{c} {\dot{Z}}_{1}^{T}P_{1}Z_{1}+Z_{1}^{T}P_{1}{\dot{Z}}_{1}+{\dot{Z}}_{2}^{T}P_{2}Z_{2}+Z_{2}^{T}P_{2}{\dot{Z}}_{2}+r^{T}r+Z_{2}^{T}Z_{2}-\lambda^{2}u^{T}u-\gamma^{2}w^{T}w\leq 0 \end{array}$$

Using Hypothesis 1 and Lemma 2, we get:

(43) $$\begin{array}{c} {\left[\begin{array}{c} Z_{1}\\ Z_{2}\\ u\\ \tilde{w} \end{array}\right]}^{T}\left[\begin{array}{cccc} \Delta_{1} & C^{T}L^{T}P_{2} & 0 & P_{1}W\\ P_{2}LC & \Delta_{2} & P_{2}B & 0\\ 0 & B^{T}P_{2} & -\lambda^{2}I & 0\\ W^{T}P_{1} & 0 & 0 & -\gamma^{2}I_{2} \end{array}\right]\left[\begin{array}{c} Z_{1}\\ Z_{2}\\ u\\ \tilde{w} \end{array}\right]\leq 0 \end{array}$$

and:

(44) $$\begin{array}{ccc} \Delta_{1} & = & H\left(P_{1}\left(A-LC\right)\right)+\delta_{1}l^{2}\bar{I}+\delta_{1}^{-1}P_{1}GG^{T}P_{1}+C_{0}^{T}N_{1}^{T}N_{1}C_{0} \end{array}$$

(45) $$\begin{array}{ccc} \Delta_{2} & = & H\left(P_{2}\left(A\right)\right)+\delta_{2}l^{2}\bar{I}+\delta_{2}^{-1}P_{2}GG^{T}P_{2}+I_{4\times 4} \end{array}$$

**Remark 2.**  *The matrix $\bar{I}$ is the consequence of applying Lemma 2 and Hypothesis 2; it characterizes in which system states the nonlinearities are applied. On the other hand, the residual signal is rewritten to fit in the dimension with the expression (42), in such a way that: $C_{0}=\left[\begin{array}{cc} C & 0_{1\times 2} \end{array}\right]$ and $N_{1}^{T}=\left[\begin{array}{cc} N^{T} & 0_{2\times 1}^{T} \end{array}\right]$. Additionally, the matrix $I_{2}$ is written as follows:*

$$\begin{array}{c} I_{2}=\left[\begin{array}{cccc} 1 & 0 & 0 & 0\\ 0 & 1 & 0 & 0\\ 0 & 0 & 0 & 0\\ 0 & 0 & 0 & 0 \end{array}\right] \end{array}$$

Using the Schur Complement (Lemma 1) three times and denoting $U_{1}=P_{1}L$, $U_{2}=P_{2}L$ yields the LMI constraints of (35)–(39), and that ends the proof. □

### 2.3. Descriptor Observer Design

The descriptor observer is based on the descriptor systems approach. The idea is to assume the additive sensor faults as a system state, in such a way that the resulting augmented system represents a descriptor system. Thus, the descriptor observer tends to estimate physical system states and the additive faults thanks to the appropriate gain matrices [52].

In order to design the descriptor observer, the system (27) is rewritten in the following augmented form:

(46) $$\left\{\begin{array}{cc} E\dot{\bar{x}} & =\bar{A}\bar{x}+\bar{G}g\left(x\right)+\bar{B}u+\bar{W}w+\bar{F}{\bar{f}}_{s}\\ y & =\bar{C}\bar{x}=C_{0}\bar{x}+{\bar{f}}_{s} \end{array}\right.$$

with: $\bar{x}=\left[\begin{array}{c} x\\ f_{s} \end{array}\right]$, $E=\left[\begin{array}{cc} I & 0\\ 0 & 0 \end{array}\right]$, $\bar{A}=\left[\begin{array}{cc} A & 0\\ 0 & 0 \end{array}\right]$, $\bar{G}=\left[\begin{array}{c} G\\ 0 \end{array}\right]$, $\bar{B}=\left[\begin{array}{c} B\\ 0 \end{array}\right]$, $\bar{W}=\left[\begin{array}{c} W\\ 0 \end{array}\right]$, $\bar{F}=\left[\begin{array}{c} 0\\ I \end{array}\right]$, ${\bar{f}}_{s}=Ff_{s}$, $\bar{C}=\left[\begin{array}{cc} C & F \end{array}\right]$, and $C_{0}=\left[\begin{array}{cc} C & 0 \end{array}\right]$.

The nonlinear Lipschitz descriptor observer leading to the estimate of the system states and the sensor faults is written as follows:

(47) $$\left\{\begin{array}{cc} \bar{E}\dot{z} & =Sz+\bar{G}g\left(\hat{x}\left(t\right)\right)+\bar{B}u\left(t\right)\\ \hat{\bar{x}} & =z+Ly \end{array}\right.$$

*z* is an internal variable, $S=\left[\begin{array}{cc} A & 0\\ -C & -I \end{array}\right]$, $L=\left[\begin{array}{c} 0\\ I \end{array}\right]$, $\bar{E}=\left[\begin{array}{cc} I+\Theta C & \Theta \\ RC & R \end{array}\right]$, where Θ and *R* are chosen in such a way that $\bar{E}$ is nonsingular.

Let us define the error $e=\bar{x}-\hat{\bar{x}}$ and the free faults’ residual $r=N_{2}\left(y-\hat{y}\right)=N_{2}C_{0}e$ (where $N_{2}$ is a weighting matrix to be designed). Indeed, the following error dynamics:

(48) $$\dot{e}=\tilde{S}e+\tilde{G}\tilde{g}+\tilde{W}w$$

with $\tilde{g}=g\left(x\left(t\right)\right)-g\left(\hat{x}\left(t\right)\right)$, $\tilde{S}={\bar{E}}^{-1}S=\left[\begin{array}{cc} A+\Theta R^{-1}C & \Theta R^{-1}\\ -CA-(R^{-1}+C\Theta R^{-1})C & -R^{-1}-C\Theta R^{-1} \end{array}\right]$, $\tilde{G}={\bar{E}}^{-1}\bar{G}=\left[\begin{array}{c} G\\ -CG \end{array}\right]$, $\tilde{W}={\bar{E}}^{-1}\bar{W}=\left[\begin{array}{c} W\\ -CW \end{array}\right]$.

From (27) and (48), we have the following augmented system:

(49) $$\begin{array}{c} \left[\begin{array}{c} \dot{e}\\ \dot{x} \end{array}\right]=\left[\begin{array}{cc} \tilde{S} & 0\\ 0 & A \end{array}\right]\left[\begin{array}{c} e\\ x \end{array}\right]+\left[\begin{array}{cc} \tilde{G} & 0\\ 0 & G \end{array}\right]\left[\begin{array}{c} \tilde{g}\\ g(x) \end{array}\right]+\left[\begin{array}{c} 0\\ B \end{array}\right]u+\left[\begin{array}{c} \tilde{W}\\ W \end{array}\right]w \end{array}$$

The stability of the system (49) is ensured, using Property 1, and the $L_{2}$-gain form, if the LMI condition summarized in the following Theorem holds:

**Theorem** **3.** *The nonlinear augmented Lipschitz descriptor system is asymptotically stable, if there exist positive definite matrices ${\bar{P}}_{11}$, ${\bar{P}}_{12}$, and ${\bar{P}}_{2}$, matrices ${\bar{N}}_{1}$, ${\bar{N}}_{2}$ and $N_{2}$ and positive scalars $\bar{\lambda }$, $\bar{\gamma }$ and $\delta_{i}\left(i=3,4\right)$, such that the following LMI condition is satisfied:*

(50) $$\begin{array}{c} \left[\begin{array}{cc} \Sigma_{22} & \Upsilon_{22}\\ {}* & \Xi_{22} \end{array}\right]\leq 0 \end{array}$$

*and:*

(51) $$\begin{array}{ccc} \Sigma_{22} & = & \left[\begin{array}{ccc} \Phi_{11} & \Phi_{12} & 0\\ {}* & \Phi_{21} & 0\\ {}* & * & \Phi_{31} \end{array}\right] \end{array}$$

(52) $$\begin{array}{ccc} \Upsilon_{22} & = & \left[\begin{array}{ccccc} 0 & {\bar{P}}_{11}W & {\bar{P}}_{11}G & C^{T}N_{2}^{T} & 0\\ 0 & -P_{12}CW & -{\bar{P}}_{12}CG & 0 & 0\\ {\bar{P}}_{2}B & {\bar{P}}_{2}W & 0 & 0 & {\bar{P}}_{2}G \end{array}\right] \end{array}$$

(53) $$\begin{array}{ccc} \Xi_{22} & = & \left[\begin{array}{ccccc} -{\bar{\lambda }}^{2}I & 0 & 0 & 0 & 0\\ {}* & -{\bar{\gamma }}^{2}I & 0 & 0 & 0\\ {}* & * & -\delta_{3}^{-1}I & 0 & 0\\ {}* & * & * & -I & 0\\ {}* & * & * & * & -\delta_{4}^{-1}I \end{array}\right]\\ \Phi_{11} & = & H\left({\bar{P}}_{11}A\right)+H\left({\bar{N}}_{1}C\right)+\delta_{3}l^{2}\bar{I}+I\\ \Phi_{12} & = & {\bar{N}}_{1}-A^{T}C^{T}{\bar{P}}_{12}^{T}-C^{T}{\bar{N}}_{2}^{T}\\ \Phi_{21} & = & -H({\bar{N}}_{2})+I\\ \Phi_{31} & = & H\left({\bar{P}}_{2}A\right)+\delta_{4}l^{2}\bar{I}+I \end{array}$$

The estimated fault is written as follows:

(54) $${\hat{f}}_{s}=-{\left(F^{T}F\right)}^{-1}F^{T}{\hat{\bar{f}}}_{s}$$

where:

(55) $${\hat{\bar{f}}}_{s}=\left[\begin{array}{cc} 0 & I \end{array}\right]\hat{\bar{x}}$$

**Proof.**  Consider the following Lyapunov function, where ${\bar{P}}_{1}={\bar{P}}_{1}^{T}>0$ and ${\bar{P}}_{2}={\bar{P}}_{2}^{T}>0$ are symmetric definite positive matrices, of appropriate dimensions:

(56) $$V\left(e\left(t\right),x\left(t\right)\right)={\left[\begin{array}{c} e\\ x \end{array}\right]}^{T}\left[\begin{array}{cc} {\bar{P}}_{1} & 0\\ 0 & {\bar{P}}_{2} \end{array}\right]\left[\begin{array}{c} e\\ x \end{array}\right]\leq 0$$

Deriving (56) and using Property 1, as well as the $L_{2}$-gain form, we obtain:

(57) $$\dot{V}\left(e\left(t\right),x\left(t\right)\right)+r^{T}r+x^{T}x-{\bar{\gamma }}^{2}w^{T}w-{\bar{\lambda }}^{2}u^{T}u$$

Using Hypothesis 1 and Lemma 2, Equation (57) can be written as follows:

(58) $${\left[\begin{array}{c} e\\ x\\ u\\ d \end{array}\right]}^{T}\left[\begin{array}{cccc} \Gamma_{1} & 0 & 0 & {\bar{P}}_{1}\tilde{W}\\ {}* & \Gamma_{2} & B^{T}{\bar{P}}_{2} & {\bar{P}}_{2}W\\ {}* & * & -{\bar{\lambda }}^{2}I & 0\\ {}* & * & * & -{\bar{\gamma }}^{2}I \end{array}\right]\left[\begin{array}{c} e\\ x\\ u\\ d \end{array}\right]<0$$

with:

$$\begin{array}{ccc} \Gamma_{1} & = & H\left({\bar{P}}_{1}\tilde{S}\right)+\delta_{3}l^{2}I+\delta_{3}^{-1}{\bar{P}}_{1}\tilde{G}{\tilde{G}}^{T}{\bar{P}}_{1}+C_{0}^{T}N_{2}^{T}N_{2}C_{0}+I\\ \Gamma_{2} & = & H\left({\bar{P}}_{2}A\right)+\delta_{4}l^{2}II+\delta_{4}^{-1}{\bar{P}}_{2}GG^{T}{\bar{P}}_{2}+I \end{array}$$

Using the Schur complement twice:

(59) $$\left[\begin{array}{cccccc} \Gamma_{1}^{\prime } & 0 & 0 & {\bar{P}}_{1}\tilde{W} & {\bar{P}}_{1}\tilde{G} & C_{0}^{T}N_{2}^{T}\\ {}* & \Gamma_{2} & B^{T}{\bar{P}}_{2} & {\bar{P}}_{2}W & 0 & 0\\ {}* & * & -{\bar{\lambda }}^{2}I & 0 & 0 & 0\\ {}* & * & * & -{\bar{\gamma }}^{2}I & 0 & 0\\ {}* & * & * & * & -\delta_{3}^{-1}I & 0\\ {}* & * & * & * & * & -I \end{array}\right]<0$$

where $\Gamma_{1}^{\prime }=H\left({\bar{P}}_{1}\tilde{S}\right)+\delta_{3}l^{2}\bar{I}+I$.

Substituting $\tilde{S},\tilde{G}$ and $\tilde{W}$ by their values and taking ${\bar{P}}_{1}=\left[\begin{array}{cc} {\bar{P}}_{11} & 0\\ 0 & {\bar{P}}_{12} \end{array}\right]$, ${\bar{N}}_{1}=P_{11}\Theta R^{-1}$ and ${\bar{N}}_{2}=P_{12}\left(R^{-1}+C\Theta R^{-1}\right)$ implies:

(60) $$\left[\begin{array}{ccccccc} \Phi_{11} & \Phi_{12} & 0 & 0 & {\bar{P}}_{11}W & {\bar{P}}_{11}G & C^{T}N_{2}^{T}\\ {}* & \Phi_{21} & 0 & 0 & 0 & 0 & 0\\ {}* & * & \Gamma_{2} & B^{T}{\bar{P}}_{2} & {\bar{P}}_{2}W & 0 & 0\\ {}* & * & * & -{\bar{\lambda }}^{2}I & 0 & 0 & 0\\ {}* & * & * & * & -{\bar{\gamma }}^{2}I & 0 & 0\\ {}* & * & * & * & * & -\delta_{3}^{-1}I & 0\\ {}* & * & * & * & * & * & -I \end{array}\right]<0$$

Using the Schur complement yields the LMI constraint of (50). □

## 3. Experimental Bench

**Figure 3:**
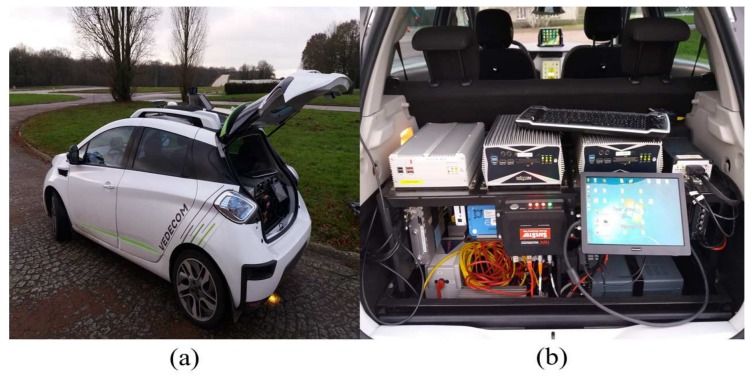
(**a**) The VEDECOMdemonstrator. (**b**) Hardware platform of the vehicle demonstrator.

This section is devoted to the evaluation of the proposed fault tolerant scheme through a real driving scenario data. For that purpose, the VEDECOM demonstrator is used (you can refer to Figure 3). This demonstrator, is a bi-mode electric and connected vehicle. Further, it is based on a Renault Zoe electric vehicle, and equipped by VEDECOM teams with several autonomous requirements equipments as Lidar (Velodyne VLP-16, VELODYNE LIDAR Inc., San Jose, CA, USA), Radar (Continental ARS-3XX series, CONTINENTAL AG, Hanover, Germany), GPS-RTK sensors and a DSpace MicroAutoBox (MABx) real time embedded computer. The wheel speeds, the motor speed and the steering wheel angle are measured by the sensors embedded in the vehicle architecture for ABS (Anti-lock Brake System) and ESP (Electronic Stability Program).

**Figure 4:**
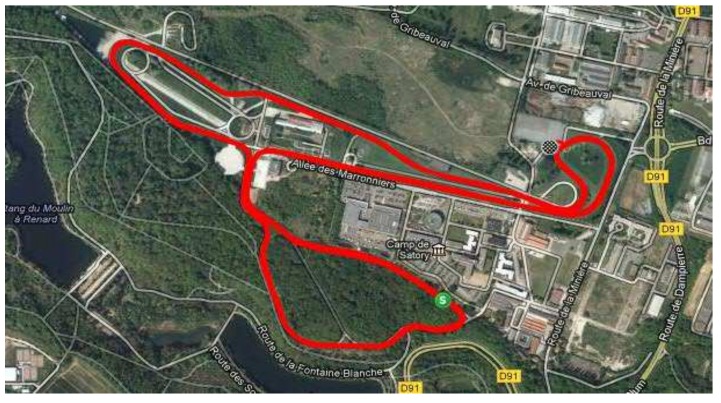
Aerial view of the Satory test track.

The experiments were conducted on the Satory test track (see Figure 4). The approach consists of self-driving mode, obeying a given reference speed profile, and in addition, recording the measured speed, the acceleration and braking torques of the vehicle, thanks to the CAN bus. This experimental data help us to validate the proposed fault tolerant scheme. Proceeding in this way allows us to properly evaluate our FTC proposition.

Indeed, this methodology provides us the assurance that the lateral dynamics (which has not been taken into account in the model dynamics) do not influence the longitudinal speed and fault estimations.

The fault tolerant strategies are tested in the real-time software RTMaps (See: https://intempora.com/products/rtmaps.html for more details), which is a modular toolkit for multi-modal applications and provides simplicity to test and validate ADAS and autonomous driving applications. Thus, RTMaps modules of the observers are build from the MATLAB/Simulink scheme. The build task is made with respect to the sampling time of the sensor measurements; to this end, the C++ compilation builder and the Simulink blocks are set to the same sampling time of the measurement logged data, in our case 10 ms.

Nevertheless, before the building of the modules, the LMI conditions of Theorems 2 and 3 are solved in order to obtain the SOF controller DO and PIO gains. These gains, as well as the Lyapunov matrices are given in Appendix A.

## 4. Experimental Results and Discussions

### 4.1. Descriptor Observer Results

We want, through this manoeuvre, to give an outline of the autonomous vehicle driving in the case of stop and go. Initially, the vehicle has a velocity of 1 m/s. Then, the vehicle accelerates to reach the speed of 5 m/s. At t=60 s, the vehicle carries out a deceleration until stopping, and thereafter, at t=80 s, the vehicle accelerates to reach a velocity of 10 m/s at t=110 s. On can note that both estimated and measured speed are identical, which proves the convergence of the observer in a finite time with a negligible steady state error.

**Figure 5:**
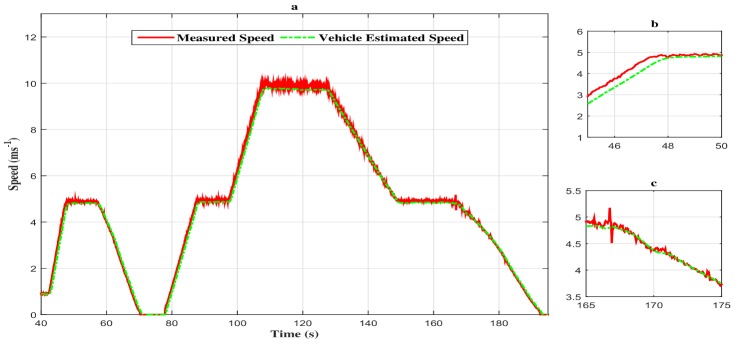
(**a**) Speed profile of the vehicle with the descriptor observer. (**b**) Zoom in at t∈[45s,50s]. (**c**) Zoom in at t∈[165s,175s].

**Figure 6:**
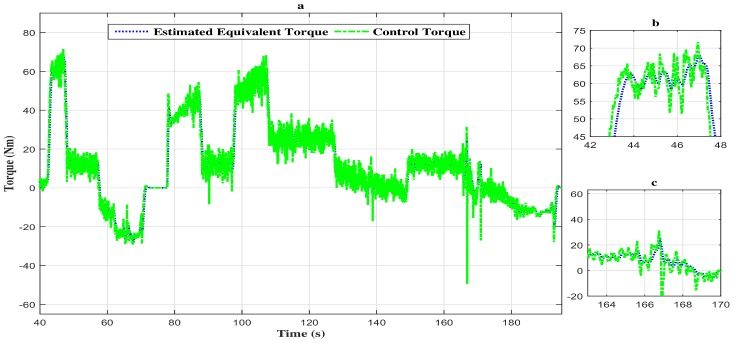
(**a**) Torque evolution of the vehicle with the descriptor observer closed loop FTC. (**b**) Zoom in at t∈[42s,48s]. (**c**) Zoom in at t∈[163s,170s].

**Figure 7:**
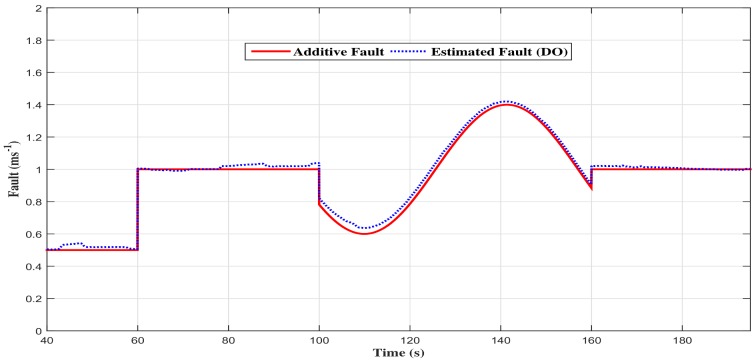
The additive fault with the descriptor observer closed loop FTC.

As we can notice through Figure 5, Figure 6 and Figure 7, the measured signal and the estimated one are simultaneously represented. The following remarks can be deduced:

- The estimated states (speed, equivalent torque and fault) converge quickly toward the real states;
- The performances obtained are good in dynamic, as well as in static output;
- The observation errors are steered to zero in finite time;
- The estimated vehicle speed seems to be insensitive to the fault variation and, so, in different phases of the considered driving scenario (accelerating phase, decelerating phase and constant speed phase).

Moreover, the estimated torque tracks the vehicle control torque with a high attenuation level of the disturbances as depicted in Figure 6a–c.

### 4.2. Proportional and Integral Observer Results

The proportional and integral observer is tested in identical conditions as previously in terms of driving manoeuvre and fault type.

**Figure 8:**
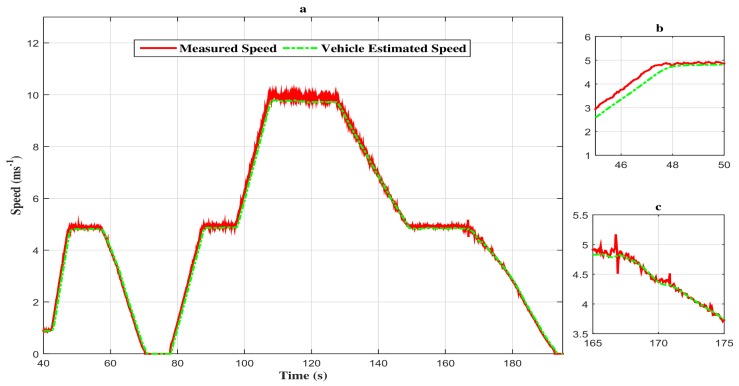
(**a**) Speed profile of the vehicle with the proportional and integral observer closed loop FTC. (**b**) Zoom in at t∈[45s,50s]. (**c**) Zoom in at t∈[165s,175s].

Thus, as depicted in Figure 8a–c, the estimated vehicle speed tracks the measured vehicle speed with attenuation of the disturbances.

**Figure 9:**
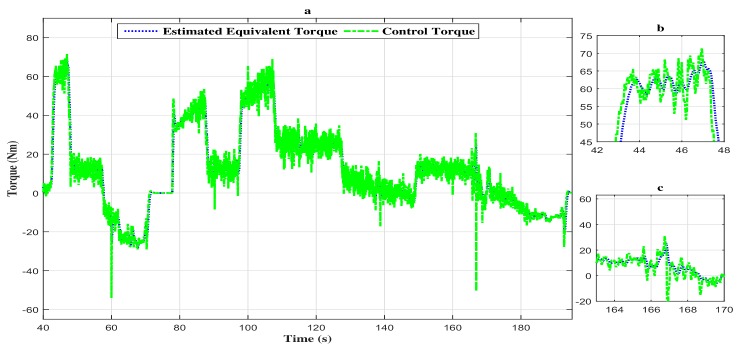
(**a**) Torque evolution of the vehicle with the proportional and integral observer closed loop FTC. (**b**) Zoom in at t∈[42s,48s]. (**c**) Zoom in at t∈[163s,170s].

**Figure 10:**
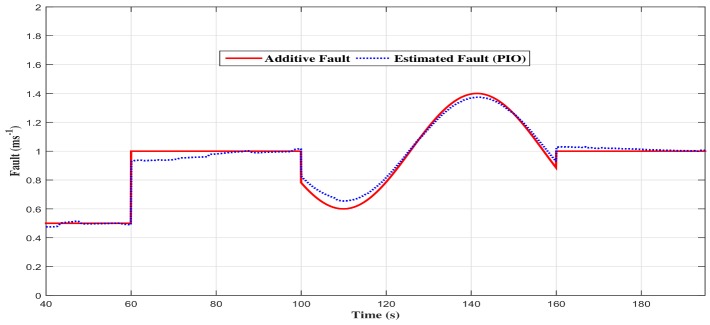
The additive fault with the proportional and integral observer.

Additionally, the estimated torque with the proportional and integral observer converges to the control torque (see Figure 9a–c). Furthermore, the estimated fault with the proportional and integral observer struggles to converge in the event of abrupt additive fault variations. In fact, the variations at t= 60 s, t= 100 s and t= 160 s generate a small fault estimation error that needs time to be cancelled (see Figure 10).

### 4.3. Comparison of the Two Observers

Globally, the two observers have shown a high ability to detect and estimate the additive sensors faults, since the separation principle is applied to both observers and the same control approach is designed based on Lyapunov theory.

**Figure 11:**
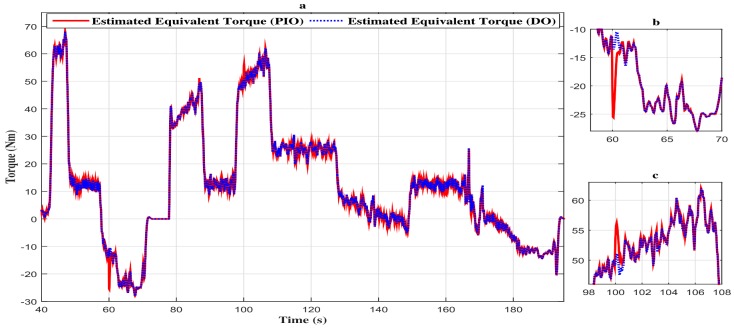
(**a**) Comparison of the vehicle estimated torque with the two observers. (**b**) Zoom in at t∈[58s,70s]. (**c**) Zoom in at t∈[98s,108s]

In fact, the estimated equivalent torque comparison between descriptor observer and proportional integral observer is depicted in Figure 11a. From this figure, we can note that the descriptor observer has a significant disturbance attenuation level compared to the proportional and integral observer. Further, we can notice that the abrupt additive fault variations affect the estimated equivalent torque, as shown in Figure 11b,c. Additionally, estimation error is very small with the proportional and integral observer and negligible with the descriptor observer.

**Figure 12:**
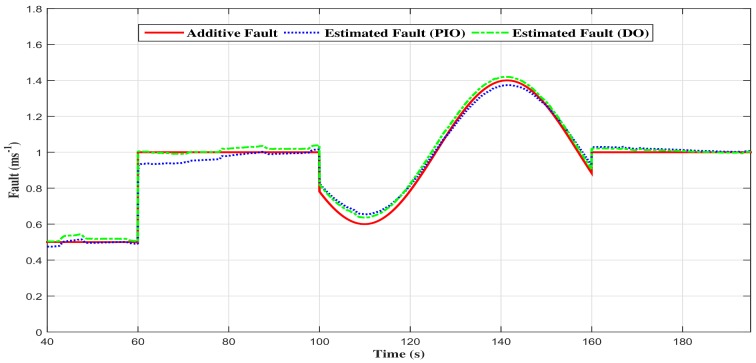
Comparison of the estimated additive fault with the two observers.

**Figure 13:**
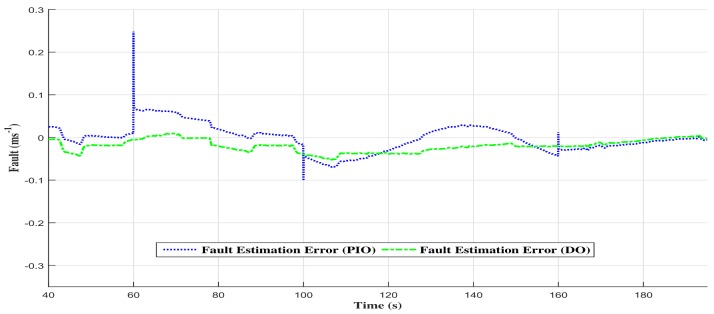
Comparison of fault estimation error between the two observers.

In fact, Figure 12 shows the comparison between the two observers fault estimations, and the Figure 13 shows the comparison of the fault estimation error. One can notice that the descriptor observer estimated fault state tracks the emulated one where the performances are good in dynamic, as well as in static output. On the other hand, the proportional and integral observer seems to present a small estimation error. This error is the consequence of the abrupt variations in the additive fault, and it is due to the proportional and integral observer scheme.

**Figure 14:**
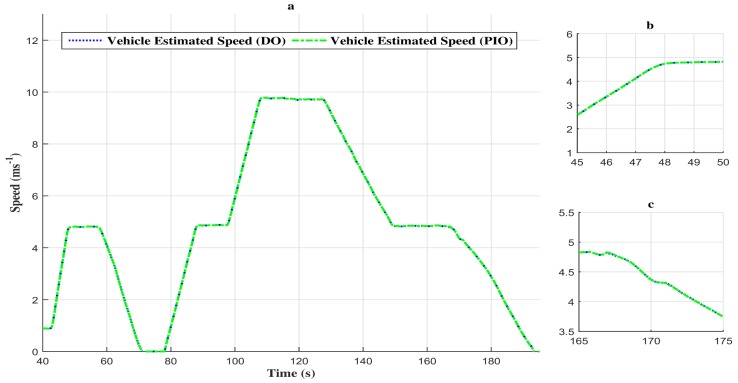
(**a**) Comparison of the vehicle estimated speed with the two observers. (**b**) Zoom in at t∈[45s,50s]. (**c**) Zoom in at t∈[165s,175s].

**Figure 15:**
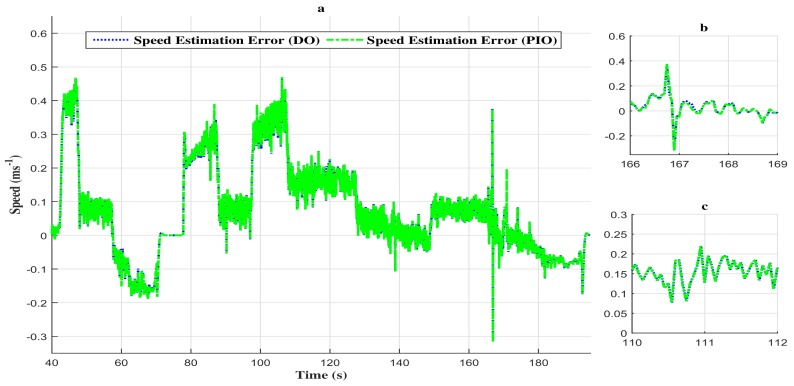
(**a**) Comparison of speed estimation error between the two observers. (**b**) Zoom in at t∈[166s,169s]. (**c**) Zoom in at t∈[110s,112s]

The difference between the descriptor observer and the proportional and integral observer in performing fault estimation is due to the nature of the last one. In fact, in a real-time environment, the numerical integration may not be achievable and lead to a highly time-consuming process, thus generating significant estimation errors. However, the vehicle speed estimation by the two observers presents no significant difference (see Figure 14a–c). Indeed, the speed estimation error comparison given in Figure 15 shows a negligible estimation error (around 0.4 ms${}^{-1}$).

### 4.4. FTC Results

In order to test the proposed FTC scheme, the closed-loop approach is designed by numerical simulations using MATLAB/Simulink. Furthermore, the proposed adaptive cruise control FTC (depicted in Figure 1) is simulated with the same scenarios of the speed profile and fault.

**Figure 16:**
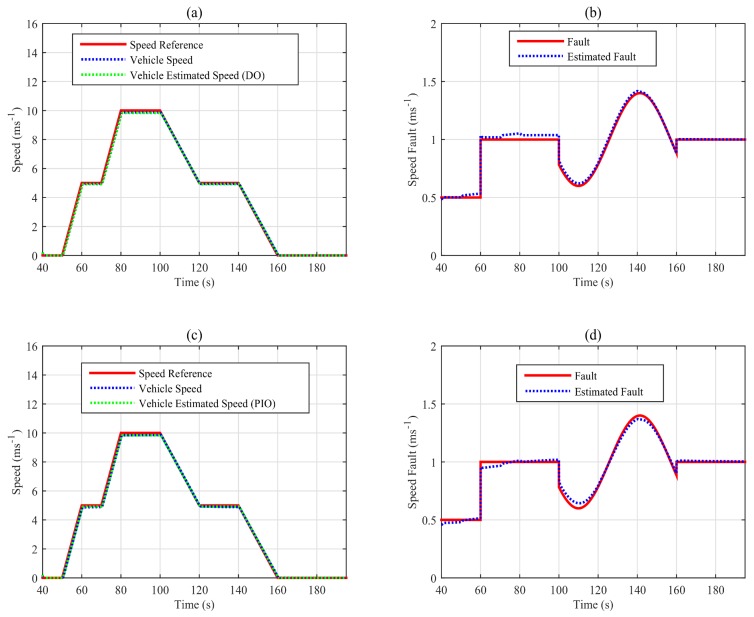
Speed profile( (**a**) with descriptor observer, (**c**) with proportional and integral observer) and fault estimations ( (**b**) with descriptor observer, (**d**) with proportional and integral observer) of the simulated closed-loop FTC scheme.

Indeed, Figure 16a shows the speed profile of the vehicle in blue, which tracks the reference speed in red with the descriptor observer closed-loop FTC. In fact, the vehicle speed is well estimated as shown in the figure (the green line). In addition, the estimation of the additive fault is given in Figure 16b, where we can see a good estimation. On the other hand, the speed profile of the closed-loop FTC with the proportional and integral observer is shown in Figure 16c. The designed static output feedback control shows the good tracking performance of the reference speed. Indeed, the estimated speed converges to the estimated one. Figure 16d shows the estimation of the additive fault given by the proportional and integral observer.

Simulations have been carried out to illustrate the ability of this approach, to give the good performance of the states’ estimation and FTC control scheme design in scenarios of autonomous driving. From this prospect, the tests on the vehicle prototype will be implemented.

## 5. Conclusions

The purpose of the proposed study is the sensor fault tolerant design of an autonomous vehicle. The designed theory is based on the separation principle. This approach consists of the design, in a separate manner, of a controller (a static output feedback) and a sensor fault estimation observer (a descriptor and a proportional and integral observer). Indeed, this methodology is easy to implement where the controller and observers are considered in a convex LMI optimization problem, avoiding in recourse to the use of the complex Bilinear Matrix Inequalities (BMI) in the case of an observer-based controller concept design. The experimental results of the proposed scheme show a high ability in estimating the additive fault and in maintaining a safe operating behaviour. Additionally, the designed observers have accurately estimated unmeasurable vehicle states (the vehicle equivalent torque), and this ability may be interesting when we do not need to measure a vehicle side slip angle for example or avoiding the design of cascading observers; this is made possible by the Lipschitz model used in this designed control approach. Thus, all vehicle dynamics that can be in Lipschitzian form are eligible for the method studied in this paper. The obtained successful experimental results will represent the basis of our future works in the design of fault detection for vehicle exteroceptive sensors (such as radars, LiDARs and cameras) and where the accurate proprioceptive informations must be highly accurate and fault tolerant. Afterwards, the robustness against parameter uncertainties will be taken into account; thus, the vehicle will be able to operate in all conditions.

## Figures and Tables

**Table 1:** Vehicle parameters.

Notation	Definition	Unit
*m*	Vehicle mass	kg
$V_{x}$	Vehicle speed	ms${}^{-1}$
$F_{x_{i}}$	Tire/road force of the *i*-th wheel	N
$F_{a}$	Aerodynamic force	N
${\bar{J}}_{r}$	Global inertia of the front axle	kg·m${}^{2}$
${\bar{J}}_{r_{i}}$	Inertia of the *i*-th front wheel	kg·m${}^{2}$
${\dot{\omega }}_{r_{i}}$	Acceleration of the *i*-th wheel	rad·s${}^{-2}$
$T_{m}$	The engine torque	Nm
*r*	The tire radius	m
$F_{r_{i}}$	The rolling force of the *i*-th wheel	N
$T_{b_{i}}$	The braking torque of the *i*-th wheel	Nm
$T_{r_{f}}/T_{r_{r}}$	The rolling torque of the front/rear axle	Nm
$J_{r}$	Global inertia of the rear axle	kg·m${}^{2}$
$J_{r_{i}}$	Inertia of the *i*-th rear wheel	kg·m${}^{2}$

## Abbreviations

The following abbreviations are used in this manuscript:

SAE	Society of Automotive Engineers
ADAS	Advanced Driver-Assistance Systems
FDD	Fault Detection and Diagnosis
FTC	Fault Tolerant Control
LMI	Linear Matrix Inequality
BMI	Bilinear Matrix Inequality
MABx	MicroAutoBox
LTI	Linear Time Invariant
DO	Descriptor Observer
PIO	Proportional and Integral Observer
SOF	Static Output Feedback

## Appendix A. The Different Gain Matrices

To solve the LMI conditions of Algorithm 1 and Theorems 2 and 3, we use the penlab solver running under the yalmpi environment of MATLAB. The LMI solutions are given as follows:

- The static output feedback controller:
  The optimization is run for an objective of ϵ=0.1 and took eight steps.
  The state feedback gain is obtained with a $H_{\infty }$ criterion of $\gamma_{01}=0.7071$, $\delta_{01}=-1.4758\times 10^{4}$ and matrices: $K_{\infty 1}=\left[\begin{array}{cc} 751.2876 & 22.5961 \end{array}\right]$, $K_{\infty 2}=\left[\begin{array}{cc} -165.1174 & -35.3645 \end{array}\right]$, $Y=10^{3}*\left[\begin{array}{cc} 1.3924 & 0.0005 \end{array}\right]$ and $X_{\infty }=\left[\begin{array}{cc} 0.5 & 0\\ 0 & 0.5 \end{array}\right]$.
  Thus, the initializing state feedback gain is given:
  $K_{SF}=YX_{\infty }^{-1}=10^{3}\left[\begin{array}{cc} 2.7847 & 0.0011 \end{array}\right]$.
  At the end of the algorithm, the $H_{\infty }$ criterion of the the two parts is given: $\gamma_{00}^{part\;1}=1.0087$, $\gamma_{00}^{part\;2}=1.0857$.
  The the static output feedback control gain is given:
  $K_{SOF}=-19.0598$.
- The proportional and integral observer:
  For an $H_{\infty }$ criterion of γ=0.1484 with a $L_{2}$ gain norm of λ=0.1266, the proportional and integral gains are given by:
  $L_{p}=\left[\begin{array}{c} -2.1481\\ 2.5996 \end{array}\right]$, $L_{I}=\left[\begin{array}{c} 108.1434\\ 15.7486 \end{array}\right]$,
  with the following positive semi-definite matrices:
  $P_{1}=\left[\begin{array}{cccc} 0.2900 & -0.0532 & 0.0335 & -0.0283\\ -0.0532 & 0.2859 & -0.0382 & 0.0225\\ 0.0335 & -0.0382 & 0.1614 & -0.0425\\ -0.0283 & 0.0225 & -0.0425 & 0.2592 \end{array}\right]$, $P_{2}=\left[\begin{array}{cccc} 0.0706 & 0.0564 & -0.0483 & 0.0281\\ 0.0564 & 0.2008 & 0.0145 & 0.2503\\ 0.0483 & 0.0145 & 0.1369 & -0.1244\\ 0.0281 & 0.2503 & -0.1244 & 0.5916 \end{array}\right]$,
  and:
  $\delta_{1}=-0.0067$, $\delta_{2}=1.7229$. $U_{1}=\left[\begin{array}{c} 2.2977\\ -2.8020\\ 16.5488\\ -0.4101 \end{array}\right]$, $U_{2}=\left[\begin{array}{c} -4.7908\\ 5.9063\\ 12.9925\\ -3.5437 \end{array}\right]$.
- The descriptor observer:
  For an $H_{\infty }$ criterion of $\bar{\gamma }=0.0377$ with a $L_{2}$ gain norm of $\bar{\lambda }=0.0811$, the descriptor gain is given by:
  $R=10^{3}\times \left[\begin{array}{cc} -0.5974 & 1.3706\\ 1.3685 & -0.6446 \end{array}\right]$, $\Theta =\left[\begin{array}{cc} -0.4480 & -0.0088\\ 0.0271 & -0.4633 \end{array}\right]$,
  so that:
  $\bar{E}=10^{3}\times \left[\begin{array}{cccc} 0.0006 & -0.0000 & -0.0004 & -0.0000\\ 0.0000 & 0.0005 & 0.0000 & -0.0005\\ -0.5974 & 1.3706 & -0.5974 & 1.3706\\ 1.3685 & -0.6446 & 1.3685 & -0.6446 \end{array}\right]$,
  with the following positive semi definite matrices:
  ${\bar{P}}_{11}=10^{3}\times \left[\begin{array}{cc} 7.8343 & 0.3447\\ 0.3447 & 7.8344 \end{array}\right]$, ${\bar{P}}_{12}=10^{3}\times \left[\begin{array}{cc} 7.8345 & 0.3448\\ 0.3448 & 7.8344 \end{array}\right]$, ${\bar{P}}_{2}=10^{3}\times \left[\begin{array}{cc} 7.8197 & 0.2733\\ 0.2733 & 7.8199 \end{array}\right]$,
  with:
  ${\bar{N}}_{1}=\left[\begin{array}{cc} -1.7233 & -3.3102\\ -3.3102 & -1.4026 \end{array}\right]$, ${\bar{N}}_{2}=\left[\begin{array}{cc} 1.9811 & 4.0318\\ -4.0318 & 2.0544 \end{array}\right]$, $N_{2}=\left[\begin{array}{cc} 0.9799 & 0\\ 0 & 1.0011 \end{array}\right]$, $\delta_{3}=1.0010$, $\delta_{4}=1.0100$.
